# Determination of trace amount of iron cations using electrochemical methods at N, S doped GQD modified electrode

**DOI:** 10.1038/s41598-023-28872-x

**Published:** 2023-01-27

**Authors:** S. Kalhori, F. Ahour, P. Aurang

**Affiliations:** 1 Department of Nanotechnology, Faculty of Chemistry, Urmia University, Urmia, Iran; 2 Institute of Nanotechnology, Urmia University, Urmia, Iran

**Keywords:** Chemistry, Nanoscience and technology

## Abstract

In this work, nitrogen and sulfur co-doped graphene quantum dot-modified glassy carbon electrodes (N, S-GQD/GCE) were used for the recognition of iron cations in aqueous solutions. The dissolved cations are detected based on the faradaic reduction or oxidation current of Fe(III) and Fe(II) obtained at the N, S-GQD/GCE surface. Cyclic voltammetry (CV), square wave voltammetry (SWV), and hydrodynamic amperometry are used as suitable electrochemical techniques for studying electrochemical behavior and determination of Fe cations. Based on the obtained results, it is concluded that the presence of free electrons in the structure of N, S-GQD could facilitate electron transfer reaction between Fe(III) and electrode surface which with increased surface area results in increased sensitivity and lower limit of detection. By performing suitable experiments, the best condition for preparing the modified electrode and determining Fe(III) was selected. Under optimized conditions, the amperometric response is linear from 1 to 100 nM of Fe(III) with a detection limit of 0.23 nM. The validity of the method and applicability of the sensor is successfully tested by the determination of Fe(III) in drug and water real samples. This sensor opened a new platform based on doped nanoparticles for highly sensitive and selective detection of analytes.

## Introduction

One of the most important elements in biological systems, environment, industry, and medicine is iron, which plays an essential role in oxygen transport, immunity, growth regulation and cell differentiation in the human body. However, excessive amounts of this ion can lead to poisoning and even death. Therefore, accurate and rapid determination of Fe(III) in environmental and biological samples is of great importance^1–3^.

Therefore, new methods have been used to achieve better detection limit (DL), better selectivity and sensitivity. Numerous analytical methods such as precipitation, spectrometry, and electrochemical sensors are available to determine iron in real samples^3–23^. Electrochemical techniques are superior to other methods for detecting Fe^3+^ due to their rapid response, high sensitivity and simplicity, and capability to miniaturization^13^. In the case of iron detection, an alternative is offered by the possibility to use the one electron oxidation of Fe(III) at solid electrodes, according to: $Fe^{3 + } + \overline{e} \leftrightarrow Fe^{2 + }$. This reaction has been exploited also for the simultaneous determination of ferrous and ferric ions^12,24–26^, but its application for practical purposes was limited to analysis at relatively high (mM) concentrations and sensitivity could be improved by using suitable mediator.

One of the important components in electrochemical methods is the working electrode, the modification of which with a suitable modifier can greatly improve the performance compared to the bare electrode^27,28^. For the development of iron sensors and biosensors, organic and biological ligands with pyridine, carboxylate, phosphate, and hydroxamate functional groups are interesting detection systems in analytical chemistry.

**Figure 1:**
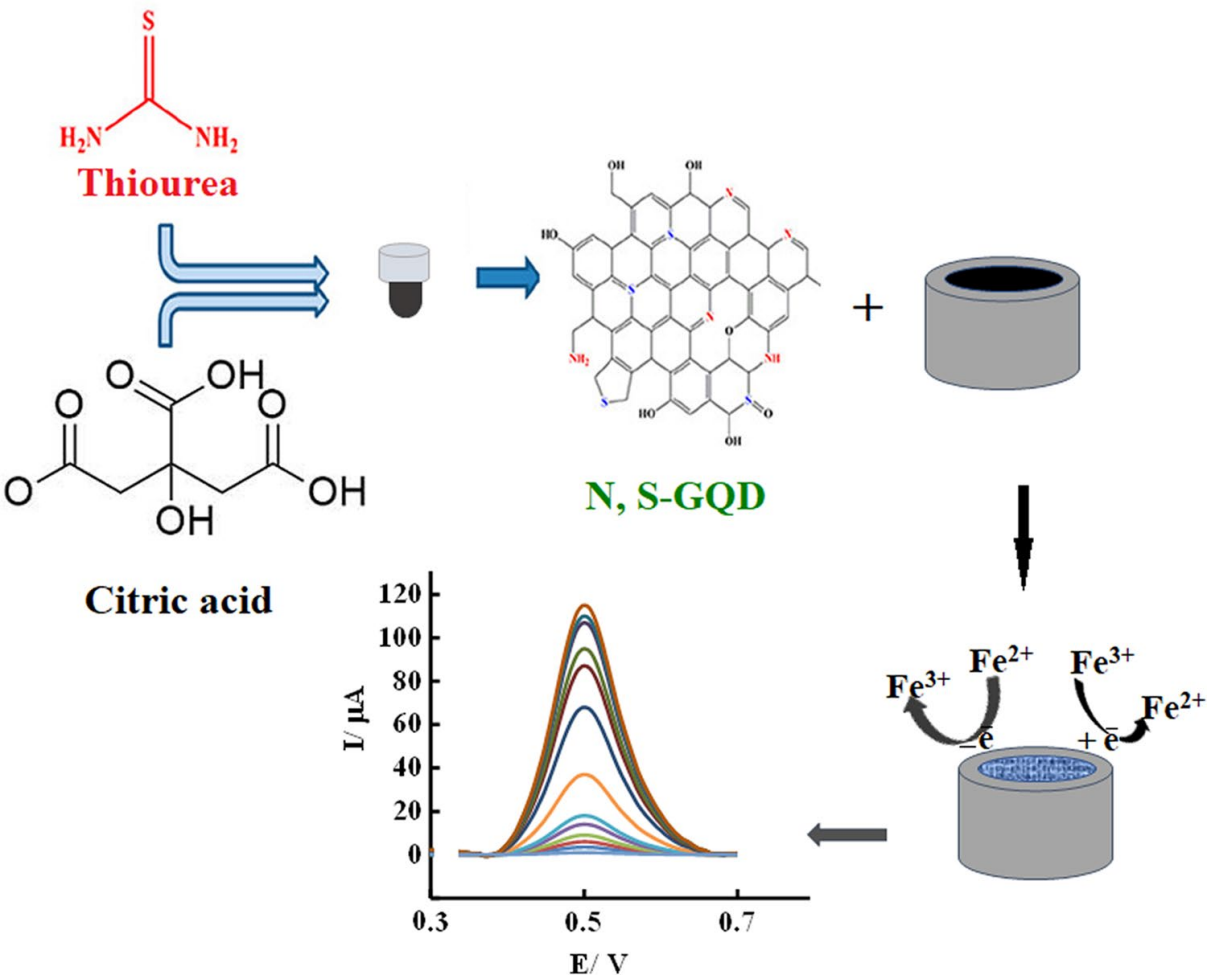
Schematic illustration of synthesis and application of N, S-GQD in Fe ions detection.

Doped graphene materials are useful in enhancing the performance of electrochemical sensors. This is because they promote charge transfer, adsorption and activation of analytes, and anchoring of functional molecules by introducing electrochemically active sites through heteroatom doping. These materials also eliminate the need of recognition elements or mediators, providing affordable and stable sensors^29^. The doping and co-doping of GQDs generates additional coordination sites and provides more defects in the structure of prepared GQDs. This nanomaterial has attractive properties which cause its wide application in fluorescent and optical sensors^30,31^. Indeed, the unique architecture and both ultrafast electron transfer and electrolyte transport by using this nano material make it as attractive electrode modifier^31–34^. Mahmoud et al. used hybrid nanomaterial of N, S-GQDs and nanocellulose for the electrochemical sensing of antischizophrenic drug. Kulandaiswamy et al. used N, S-GQDs as enzyme mimick for electrochemical Sensing of monocrotophos. Mahmoud et al. fabricated dual-mode sensor (Fluorometric and electrochemical) for the detection of toxic flavonoid rutin based on N, S-CDs^35–37^. There are also a few works on using N, S-GQDs in biosensors, hydrogen storage, or supercapacitors^38–41^. Based on the above, we used N, S-GQDs modified GCE for the sensitive and selective detection of Fe(III). To the best of our knowledge, despite interesting properties of N, S-GQD, this is the first report on the use of N, S-GQDs alone in electrochemical sensors for sensing purposes. As reported previously, electrochemical activity of heteroatom doped graphene quantum dot modified electrodes increases to a great extent due to increasing the surface area, surface defects, solubility, and the number of active sites. The band gap of heteroatom doped GQDs is effectively tuned and as a result their electrochemical properties get modulated, electron transfer rate and conductivity enhances^30^. Results confirmed excellent applicability of this nanomaterial in electrochemical sensors due to the unique characteristics of GQD with zero dimensions (0D), fast electron transfer and selective interaction of doped GQD with Fe ions (Fig. 1).

## Experimental

### Materials and instruments

Deionized water was used to prepare the solutions. All chemicals were purchased from Sigma-Aldrich Company. Analytical solutions were prepared using metal ion nitrate salts. IR spectra were recorded by the Nicolet FT-IR NEXUS 670 spectrometer (Thermo Scientific, USA) and used to identify the presence of doped groups in synthesized compounds. Raman measurement was done by HANDHELD RAMAN ANALYZER of RIGAKU (FIRSTGUARD model). TESCAN MIRA III scanning electron microscope and Zeiss Libra transmission electron microscope (working at 100 kV) were used to record FE-SEM and TEM images respectively. XRD instrument of a Bruker D8 ADVANCE X-ray diffractometer was used with a Cu-Kα radiation source (λ = 1.5406 Å) operating at 40 kV, 40 mA, and a scanning range of 10–80° 2θ, with a 2θ scan step of 0.015° and a step time of 0.2 s. UV–Vis spectra were recorded by WPA Biowave LifeScience UV–Vis spectrometer using quartz cuvettes with a path-length of 10 mm in H_2_O as the solvent. pH measurements were performed using a digital pH meter (HANNA 212). Ultrasonic bath (KODO model JAC1002) was used to clean the surface of GCE and prepare homogeneous suspensions from modifiers.

### N, S-GQDs preparation

For the synthesis of S, N- GQD, 0.21 g of citric acid (1 mmol) and 0.23 g of thiourea (3 mmol) were dissolved in 5 ml deionized water and stirred until a clear solution was obtained. The solution was then heated in an autoclave at 160 °C for 4 h.

By adding ethanol to the solution, the synthesized nanoparticles were collected and separated by centrifugation at 5000 rpm for 10 min. The resulting solid can be easily disperse in water and apply for electrode modification. For comparison, GQD was synthesized in the same way without the addition of thiourea.

### Electrode modification

The solution that used to modify the electrodes was made by mixing 10 mg of N, S-GQD with 10 ml 0.1 M KCl (pH 12) solution using ultrasonic bath for 5 min to form a homogeneous solution and then applied for the preparation of working electrode.

Before fixing the modifier at the electrode surface, it is necessary to clean the surface of the electrode by rubbing it with a polishing cloth, sonication for 5 min and thoroughly washing with distilled water.

Then, modification was done by tow electrochemical and casting method. Electrochemical stabilization of N, S-GQD on GCE was performed by performing 60 repetitive CV scans in the potential range of 0.0 to 1.0 V with a scan speed of 100 mV s^−1^. Electrostatic interactions between the surface and electron rich modifier may be the reason of this stabilization. In modification by casting, one drop (3 μl) of N, S-GQD solution (1 mg ml^−1^) was placed at the electrode surface and let to dry for 2 h.

### Electrochemical measurements

All electrochemical measurements were performed in AUTOLAB PGSTAT 30 equipment. The electrochemical cell consists of three electrodes containing modified GCE (2 mm diameter) as working electrode, Ag/AgCl (1 M KCl) as reference electrode, and platinum wire as the auxiliary electrode.

The Fe(CN)_6_^3−^/Fe(CN)_6_^4−^ was used as a redox probe to evaluate the surface changes and modifier immobilization at the electrode surface.

To study the electrochemical behavior of modified electrodes in Fe(II) analysis, 5 ml 0.1 M KNO_3_ (pH of 4.0) containing an appropriate amount of Fe(III) standard solution was added into the electrochemical cell and then the three-electrode system was put in it. The CVs were recorded in the potential range from − 0.8 to 0.8 V with a sweep rate of 100 mV s^−1^. The SWV experiment were as frequency of 15 Hz, an amplitude of 25 mV, a standing time of 40 s, and a step length of 4 mV. Prior to each measurement, there are no pretreatment required prior to repeated tests.

In order to perform hydrodynamic amperometry, 5 ml of electrolyte solution (0.5 M KNO_3_ solution with pH 4) was transferred to the voltammetric cell and after placing three electrode system in the cell, 0.4 V constant potential applied to the working electrode under hydrodynamic condition (constant stirring speed of 800 rpm). After stabilizing baseline current, by adding the exact volume (about 0.2 ml) of the standard solution of iron (III) to the cell, the iron concentration increases which result in the generation of the catalytic current. This analyte addition continued until the current no longer increases. Evaluation of the amount of iron(III) in the solution is done by the standard addition method.

## Results and discussion

### Characterization of N, S-GQD

**Figure 2:**
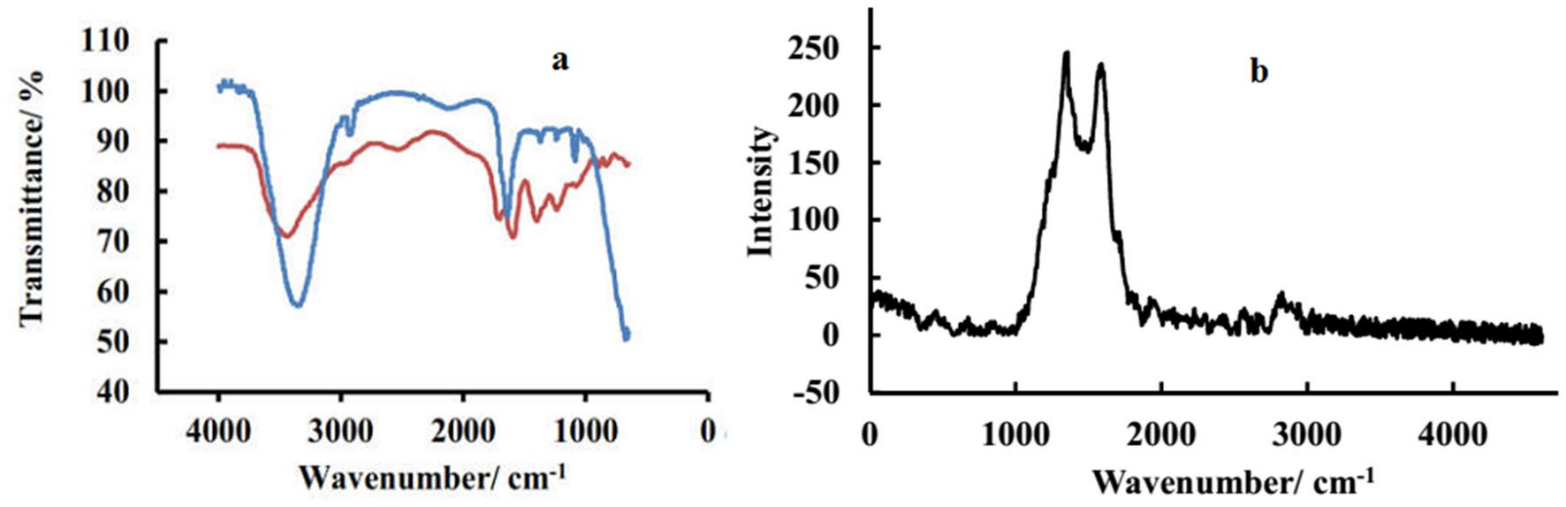
(**a**) FT-IR spectra of (red) GQD and (blue) N, S-GQD; (**b**) Raman spectrum of N, S-GQD.

FT-IR spectroscopic study was utilized to investigate the incorporation of N and S between C atoms in the GQD structure (Fig. 2a).

The spectrum of GQDs indicated broad peaks related to OH or NH at 3400 cm^−1^, C-H at 2940 cm^−1^, C=O at 1640 cm^−1^, and C=C at 1370 and 1240 cm^−1^. After doping of N and S between C atoms in the GQD, the spectrum showed variations at 1090 cm^−1^ related to C–N/C–S which obviously confirm the modification reaction.

The presence of D and G bands at 1345 and 1598 cm^−1^ in the raman spectrum obtained from N, S-GQD, confirms the existence of graphene derivatives (Fig. 2b). The high value of ID/IG (approximatly equal to 1.05) is related to the presence of N and S in the carbon skeleton and disordered structure^24^.

**Figure 3:**
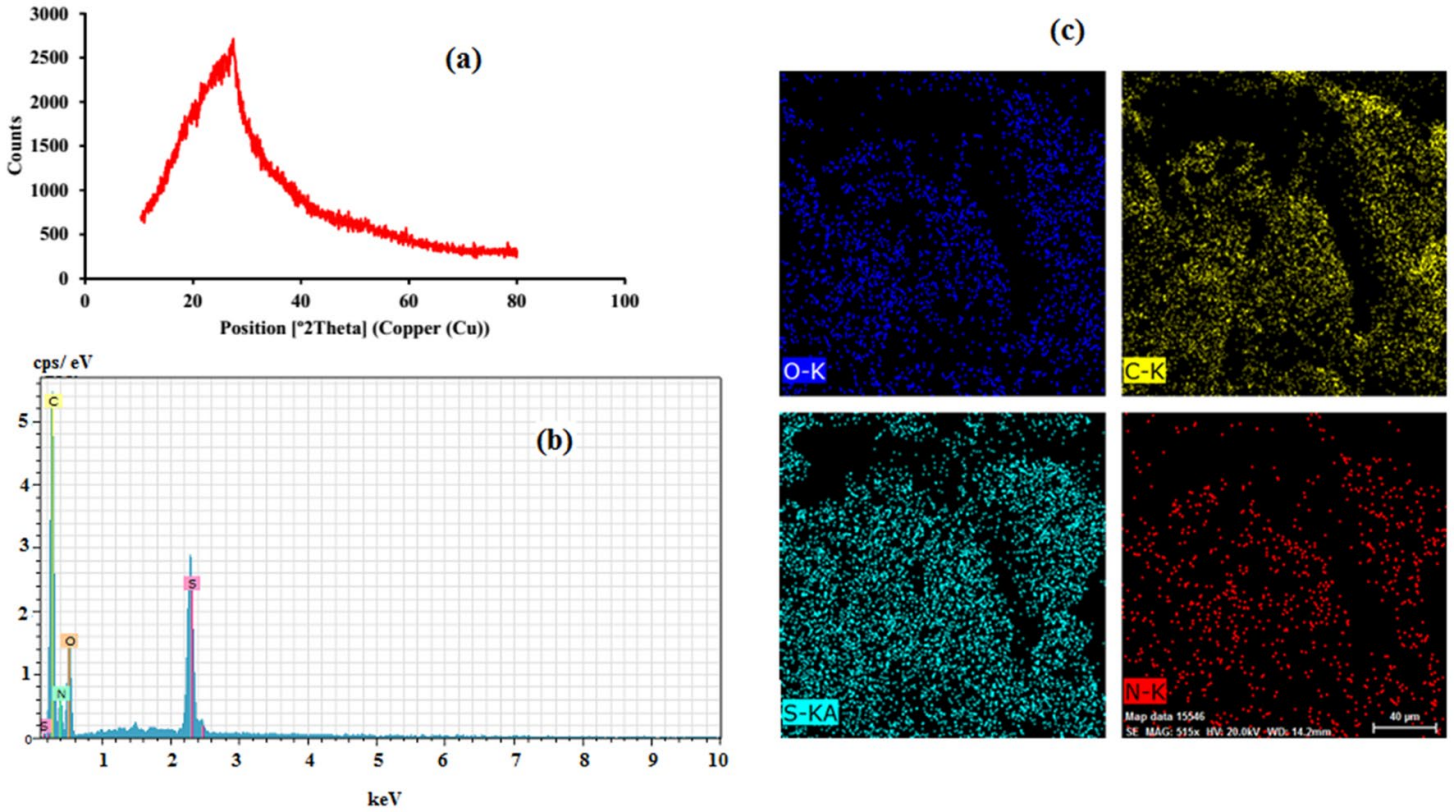
(**a**) XRD patterns of N, S-GQD; (**b**) EDX spectrum and (**c**) elemental mapping of N, S-GQD.

Figure 3a is a picture of the X-ray diffraction (XRD) of N, S-GQDs. The peaks in the picture are the places where the atoms line up in a crystal (planes in space). The peaks at 27°, are related to the (002) planes of N, S- GQDs, a new type of material that looks like graphene, but is more disordered. XRD peak broadening is related to the small size of N, S-GQDs.

Figure 3b shows a graph of the EDAX (a measure of how much energy is needed to separate the molecules in a sample). The peaks you see are for carbon, oxygen, nitrogen, and sulfur.

In order to reveal the atoms in the structure of the nanomaterial, elemental analysis was performed with an electron microscope. The appearance of peaks related to N and S along with carbon and oxygen is a confirmation of nitrogen and sulfur loading in GQD. To achieve a uniform sensor, nitrogen and sulfur doping in all quantum dots is necessary. Elemental mapping of the sample showed that almost all spots on the sample surface were doped with N and S (Fig. 3c).

**Figure 4:**
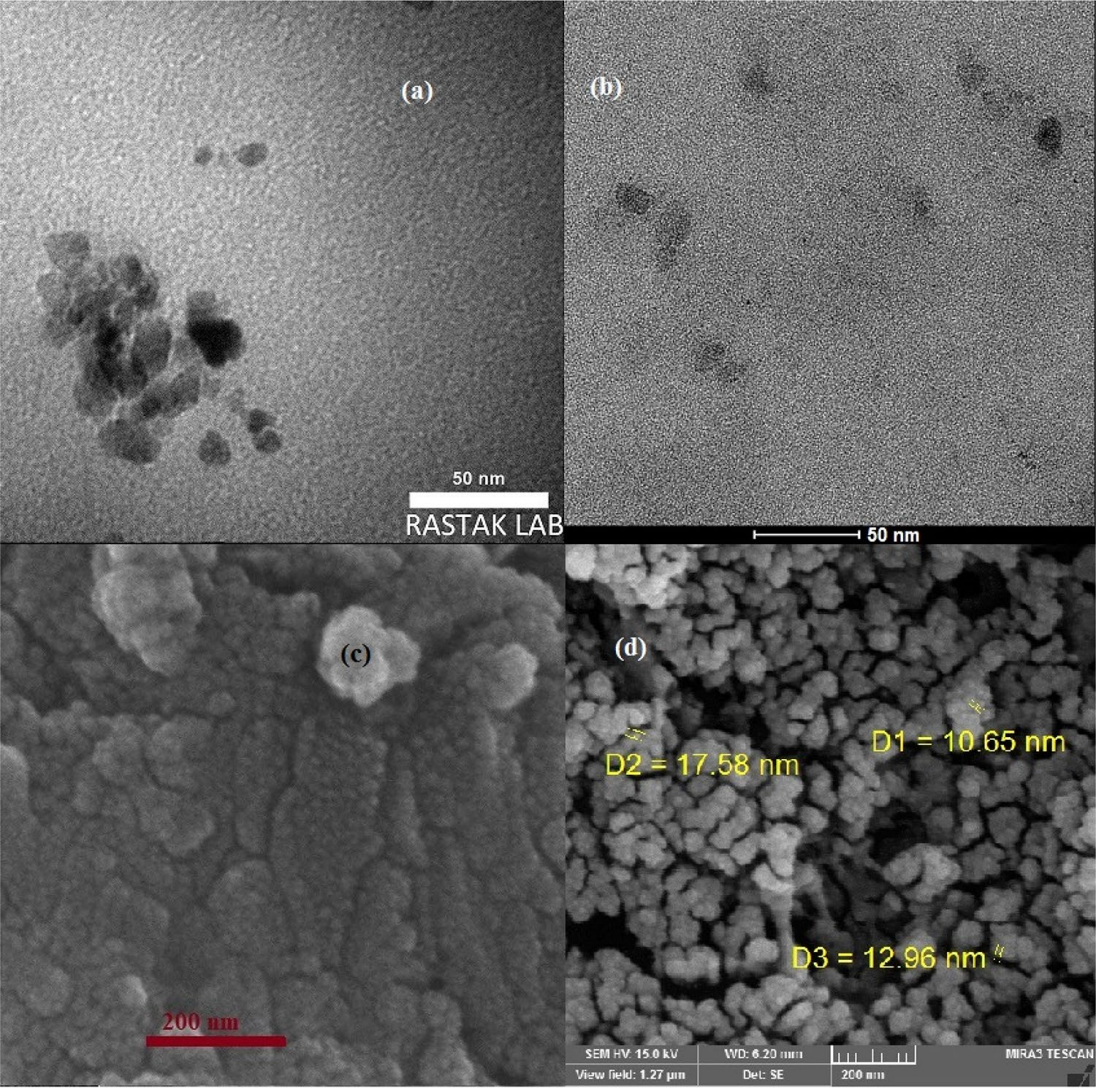
TEM (**a**), HR-TEM (**b**) and SEM (**c**) micrograph of N, S-GQD; SEM image of N, S-GQD/GCE (**d**).

The nanostructure of synthesized N, S-GQDs was characterized by TEM and SEM techniques and presented in Fig. 4.

These results show homogenous and spherical shape of synthesized nanostructure and well dispersion without agglomeration. Also, the synthesized nanoparticles were successfully stabilized on the electrode surface.

### CV experiments

For starting, unmodified and modified electrodes were placed in 5 mM solution of K_3_Fe (CN)_6_/K_4_Fe (CN)_6_ containing 0.5 M KCl and potential scanned from 0 to 1 V to asses surface area and charge transfer rate of these electrodes.

**Figure 5:**
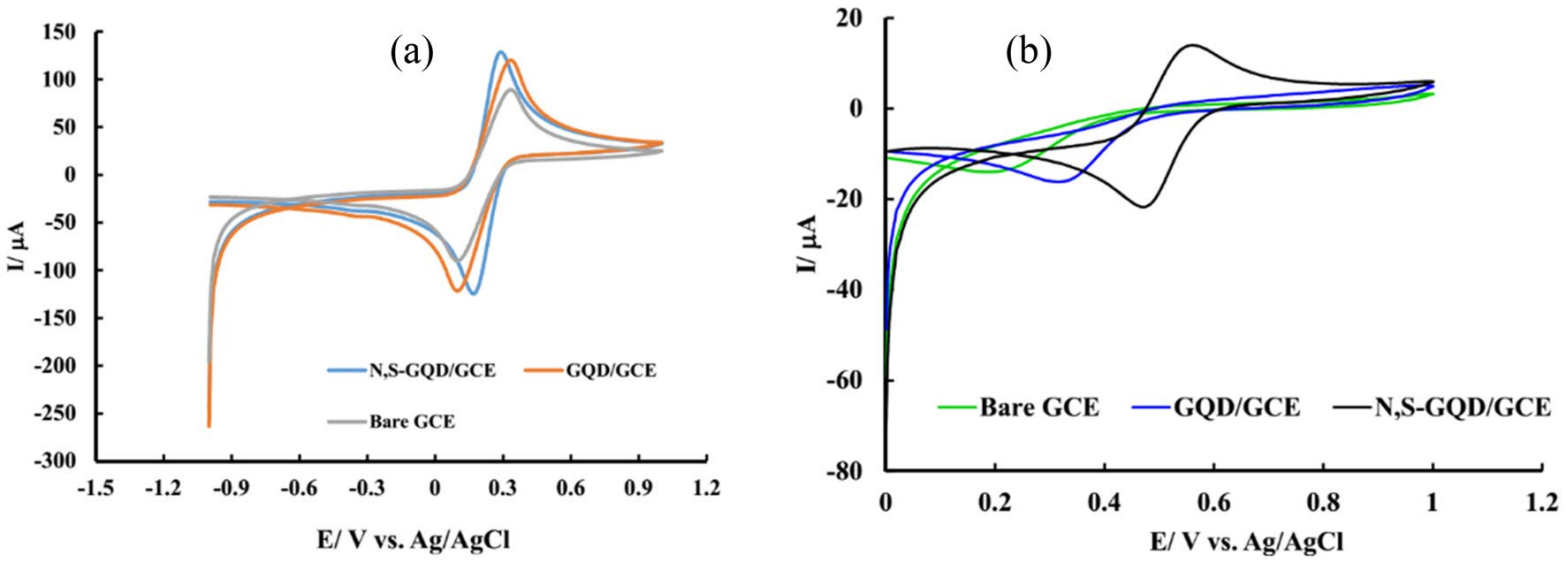
Cyclic voltammograms of bare, and modified electrodes (**a)** in 5 mM Fe(CN)_6_^3−^/Fe(CN)_6_^4−^ solution containing 0.1 M KCl and (**b**) in 0.5 M KNO_3_ containing 25 µM Fe(III).

As shown in the obtained voltammograms (Fig. 5), based on peak separation (ΔE_P_) and current values, it could be concluded that immobilization of N, S-GQD at the electrode surface accelerate charge transfer rate, increase surface area and increase obtained voltametric signal. So, applicability of the N, S-GQD/GCE in the determination of Fe(III) cations was evaluated. For this purpose, the bare and modified GCE was immersed in a 10 μM Fe(III) solution and potential scanned from 0 to 1.2 V. CV results of bare and modified electrodes were shown in Fig. 5.

As a result, bare and GQD modified GCE in this scan range cannot produce a suitable voltammetric response to Fe(III) in the CV at concentrations less than 25 µM which confirm that these electrodes surface are not suitable for electroreduction of Fe(III) or electrooxidation of Fe(II). But, a pair of well-defined and strong redox peaks appeared at the surface of N, S-GQD/GCE under the same test conditions which related to the reduction of Fe(III) to Fe(II) and consequent oxidation of produced Fe(II). The obtained excellent results related to high electrochemical activity of N, S-GQD/GCE due to increased surface area, surface defects, solubility, conductivity, and the number of active sites. It should be noted that due to the high speed of the electrochemical reaction, the direction of potential sweeping has no effect on the results and the resulting signal.

The oxidation and reduction peaks placed at 0.56 V and 0.48 V vs. Ag/AgCl, respectively. According to the published reports, the redox peaks appearing in this potential range are related to the Fe(III) redox process. The measured E_p_-E_p/2_ in reduction peak is equal to 54 mV. This is close to the theoretical value in single electron redox reaction (56.5 mV), which means that the reduction process has good reversiblity.

### Finding the best way for detection

Experimental conditions such as background electrolyte, solution pH, the amount of material that makes up the surface of the electrode and immersing time, seriously affect the detection of Fe (III) and a group of experiments were performed to adjust the effective parameters.

The amount of N, S-GQD at the electrode surface is one of the effective parameters in this sensor. For this purpose, firstly the effect of modification protocol was studied. Based on the results electrochemical modification result in higher voltametric signal for Fe(III) compared to casting (Fig. S1). In next step, effective variables in electrochemical modification such as modification scan number and scan range were studied, and optimum condition selected.

The amount of current that flows through the material changes as you change the number of cycles of the electrochemical modification. By increasing from 30 cycles, current increases, reaching a maximum at 60 cycles. Then it decreases as you keep increasing the number of modification cycles (Fig. S2). This means that at low cycles the amount of modifier is not enough to change the material, and at higher modifier value, higher resistance decreases the current.

Similarly, the effect of scan range studied, and higher signal obtained with electrode modified in the potential from 0 to 1 V which may be related to the electrostatic interactions between the modifier and electrode surface. Finally, the effect of modification scan rate studied and 100 mV s^−1^ owing to obtained signal and required time selected as optimized value (Fig. S3).

As mentioned in previous works, background electrolyte affects the signal as much as the modifier. For this purpose, three electrolyte solution containing KNO_3_, KCl, and KNO_3_ containing acetate buffer solution (ABS) used as electrolyte in the determination process. Based on the obtained results (Fig. S4), KNO_3_ and KCl are better for detection of this analyte, but in ABS electrochemical signal decreases may be due to the tendency of Fe(III) to precipitate with acetate anions. After this step, The effect of background electrolyte concentration on Fe(III) voltammetric signal was also investigated using electrolytes with concentrations ranging from 0.1 to 0.7 M. Based on the results, the concentration of 0.5 M was selected as the optimum value, which can provide sufficient conductivity for charge transfer (Fig. S5).

After choosing 0.5 M KNO_3_ as the suitable background electrolyte, the effect of pH on the resulting signal was investigated. Due to the fact that 10 μM of Fe(III) is stable at pH ≤ 4 and does not precipitate, so the effect of solution pH up to 6 was investigated. As shown in Fig. S6, as the solution gets more acidic (lower pH) or gets more basic (higher pH), the current value decreases (goes down). This result related to the decrease of charge transfer rate at very low pHs due to the protonation of free electron pair containing atoms at acidic condition and competition of hydroxide ions for reaction with iron at high pH values. Consequently, the best pH for this experiment is 4.

Based on previous reports in spectrophotometry, Fe(III) can adsorb on the N, S-GQD. Thus, in continue the effect of pre-concentration potential was studied applying defferent potential to the electrode dipped in 25 µM Fe(III) solution for 10 min. Results (Fig. S7) showed that Fe(III) can’t adsorb at the N, S-GQD/GCE, and the positive effect of N, S-GQD and high sensitivity of this sensor related to the kinetics of the charge transfer and increased surface area. It should be noted that the shape of the resulting voltammograms also confirms this.

**Figure 6:**
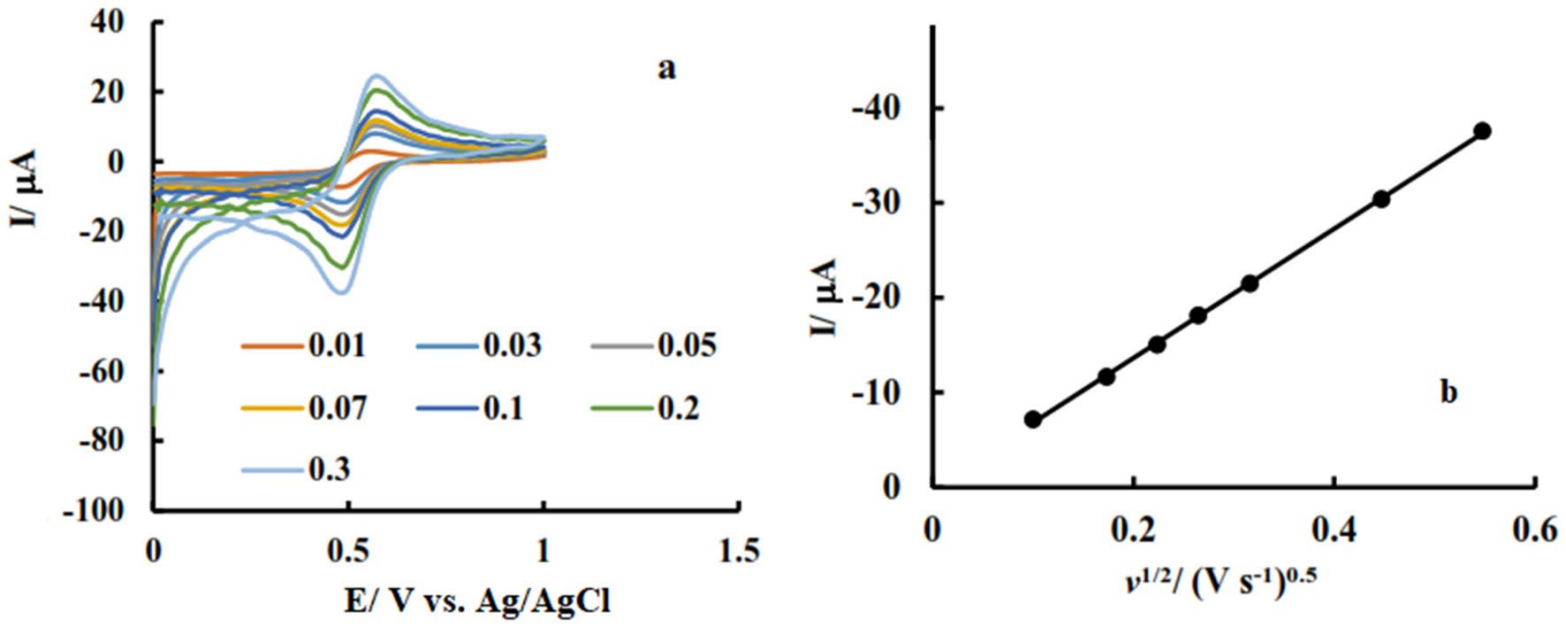
(**a**) Cyclic voltammograms of N, S-GQD/GCE in 0.5 M KNO_3_ containing 25 µM Fe(III) with various scan rates; (**b**) Variation of current versus square root of scan rate.

The effect of scan rate on the obtained voltammograms were studied and based on the results current is proportional to the square root of scan rate (Fig. 6).

This result confirm that electrochemical reaction of Fe(III) at this electrode is diffusion controlled. In addition, Fe(III) reduction peak appeared at 0.48 V vs. Ag/AgCl and increasing scan rate don’t change this value. This behavior corresponds to reversible electrochemical system and confirms good reversibility of the proposed sensor.

The effect of analyte concentration was also investigated by cyclic voltammetry. For this purpose, by adding increasing concentrations of Fe(III) to the electrochemical cell (from 3 to 40 μM) voltammetric signal recorded and the results showed that up to the concentration of 25 μM, the oxidation and reduction current has a linear relationship with the concentration and then it is level off (Fig. S8). This result confirms that obtained current is related to the Fe(III) concentration and able to detect low concentrations of iron cations applying sensitive analytical methods.

### Quantitative measurements of Fe(III)

Due to the high reversibility of the Fe(III) reduction reaction according to:$Fe^{3 + } + \overline{e} \leftrightarrow Fe^{2 + }$, determination of total amount of ferrous and ferric ions were done by square wave voltammetry (SWV).

**Figure 7:**
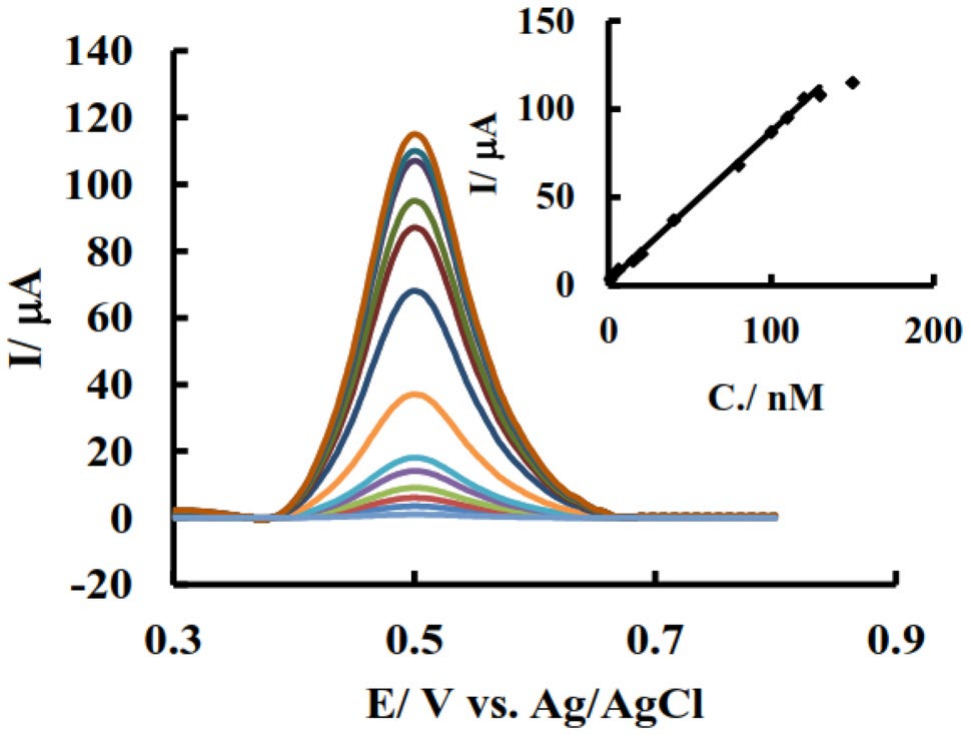
SWV of N, S-GQD in 0.5 M KNO_3_ after addition different concentrations of Fe(III); inset: Variation of current versus concentration related to above voltammograms.

Results (Fig. 7) showed that in the concentration range from 1 to 120 nM, current is proportional to the total Fe cations concentration independent from scan direction. Using this method, it is not possible to determine the individual concentration of the species because the species are immediately transformed into another one by applying potential.

**Figure 8:**
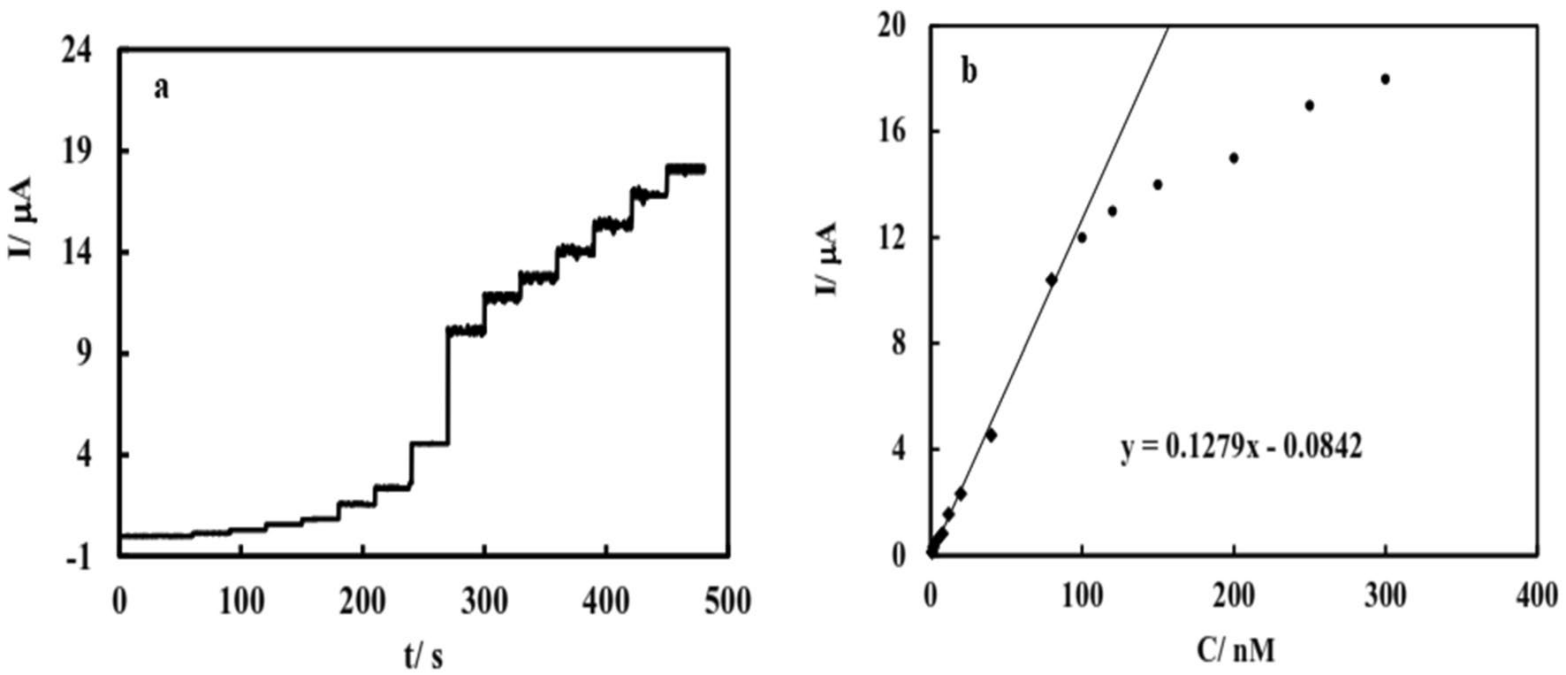
(**a**) Hydrodynamic amperograms of N, S-GQD/GCE immersed in stirred solution of KNO_3_ applying 0 V adding different amounts of Fe(III); Variation of current versus concentration obtained from presented hydrodynamic amperometry.

In continue, individual quantitative determination of Fe(III) or Fe(II) could be achieved by hydrodynamic amperometric technique, applying suitable constant potential to the N, S-GQD/GCE immersed in the stirred electrolyte solution and addition of an accurate volume of standard Fe(III) solution. Results (Fig. 8) showed that there is linear relation between concentration and current in the concentration range from 1 to 100 nM with the linear relationship obeying the following equation:

$${\text{I}}/{\mu A} = 0.128\left( {{\text{C}}/{\text{nM}}} \right) + 0.0842$$

Considering the detection limit as three times the standard deviation divided by the slope of the calibration diagram, the detection limit was calculated equal to 0.23 nM.

By reducing the concentration of iron (III) from the total amount of iron, the concentration of iron (II) in the sample can be calculated. The obtained results this sensor is comparable with other reported electrochemical sensors (Table 1).

**Table 1:** Analytical parameters of N, S-GQD/GCE compared to other modified electrodes.

Modifier	Linear range	LOD	Reference
Co-MOF*	0.1–1.2 μM	0.1 μM	^13^
TrG/NF/PDE*	17.9 nM–3.57 μM	1.42 nM	^18^
PtNF*	17.9 nM–4.46 μM	5.5 nM	^19^
rGO/AuNPs*	30 nM–3 μM	3.5 nM	^21^
TPP*/ITO*	0.1 μM–0.1 mM	0.17 μM	^42^
N, S-GQD	1–120 nM	0.23 nM	This work

*Co-MOF* Co based metal organic anion framework, *TRG/NF/PDE* Thermally reduced graphene/nafion modified platinum disk electrode, *PtNF* Nafion modified platinum electrode-on chip, *rGO/AuNPs* reduced graphene oxide/gold nanoparticles modified electrode, *TPP* Pyrene-Substituted Poly(2,5-dithienylpyrrole, *ITO* Indium tin oxide.

### Analysis of real samples

To evaluate applicability of the proposed sensor for real sample analysis, this sensor was applied for the detection of iron content in tap water and iron supplement drop (IROFANT) using standard adding method. For this purpose, 1 ml of iron drop was poured into a volumetric flask and serially diluted until its concentration stay within the range of the calibration curve. Tap water used after 1:1000 dilution without additional treatment. The amount of iron determined using SWV and hydrodynamic amperometry were presented in Table 2. Based on the results considering dilution coefficient, Fe(II) amount in IROFANT and tap water were 3.8 mM and 1.52 µM. In other words, the concentration of Fe(III) is 0.2 mM and 0.31 µM in IROFANT and tap water samples. The presence of Fe(III) in iron supplment related to oxidation of Fe(II) to Fe(III) in the presence of air oxygen and increase by passing of time.

**Table 2:** Results for the determination of Fe(III)/Fe(II) in drug and water samples.

Sample	Fe(III) added (nM)	Fe(III)/Fe(II) found (nM)	Recovery (%)	RSD (%) [a]
Iron supplement drop	0	0.2/3.8		1.9
	2	2.23/3.78	101.4	2.8
	5	5.08/3.76	97.7	3.3
Tap water	0	3.12/15.2		1.8
	3	6.22/15.16	101.6	2.1
	6	8.97/15.21	98.36	2.6

In continue, samples spiked with iron cation and the resulted solution analyzed with the proposed method. The obtained results (Table 2) and the appropriate recovery of added iron indicate the reliability of the proposed sensor.

### The reusability, stability, and selectivity of the sensor

The main thing to look for when designing a sensor is that it is reusable, stable and selective. In order to test the reusability of the sensor, the results of 20 repetitions of square wave voltammetry were compared. The N, S-GQD/GCE was refreshed by simple washing with deionized water before performing the new test, which measures the oxidation or reduction signal of iron ions. During 20 rounds of SWV tests, the curves and current intensity related to Fe ions remain constant (Fig. S9). The RSD of the obtained results in SWV are about 1.48%, which means that the proposed sensor could be used again and again without having to prepare a new one.

Then we tested how long the sensor worked. After 1 round of testing with iron nitrate (Fe(NO_3_)_3_), N, S-GQD/GCE washed and stored in the fridge (at 4 °C) for different amounts of time. Figure S10 shows the difference between the original CV curve and the one done after 1, 2 or 4 weeks later. The two curves look very similar, and 96% of the original current intensity can be kept. This means that the current is very stable and does not change much.

The selectivity has also been tested. For this, each time the first curve before addition of an interfering metal ion to the analyte solution and then after addition specific amounts of metal ions were recorded until a 5% change in the results is achieved. We chose 6 different metals including Cu(II), Pb(II), Cd(II), Zn(II), Ni(II), and Hg(II). The comparison between the initial reduction current intensity at 0.56 V for 80 nM Fe(III) and the reduction current intensity after adding different interfering ions showed that although we expected the interference of mercury in our sensor, it still works with good selectivity and results presented in Fig. S11.

## Conclusions

In this study, N, S co doped GQD was successfully synthesized and distinguished by various microscopic and optical tests. The synthesized N, S-GQD was used for the preparation of modified electrode and applied for electrochemical detection of Fe(III) and Fe(II) cations. The presence of electron rich atoms in the structure of GQD, increase surface defects and active sites, facilitate charge transfer rate and make this nanomaterial as suitable electron transferring agent to Fe cations. Using proposed sensor, the concentration of Fe(II) and Fe(III) cations were determined with good selectivity and high sensitivity. The obtained better signal at this electrode is related to charge transfer besides increased surface area and no adsorption take place which result in reusability of the modified electrode without the need for other chemicals or time-consuming processes.

## Supplementary Information

Supplementary Information.

## Data Availability

All data generated or analysed during this study are included in this published article and its supplementary information files.
