# Supplementary material for: Determination of trace amount of iron cations using electrochemical methods at N, S doped GQD modified electrode
Source: Sci Rep. 2023 Jan 27;13:1557. doi: 10.1038/s41598-023-28872-x (PMC9883219; doi:10.1038/s41598-023-28872-x)
Supplement: Supplementary file 1 — Supplementary Information. [file 41598_2023_28872_MOESM1_ESM.docx]

**Determination of Trace amount of iron cations using electrochemical methods at N, S doped GQD modified Electrode**

S. Kalhori^a^, F. Ahour^a^*, P. Aurang^a^

*^a^* Department of Nanochemistry, Faculty of science, Urmia University, Urmia, Iran

Corresponding author:

* Fatemeh Ahour

E-mail: [Fatemeh.ahour@gmail.com](mailto:Fatemeh.ahour@gmail.com), f.ahour@urmia.ac.ir; Fax: +98 44-32752746


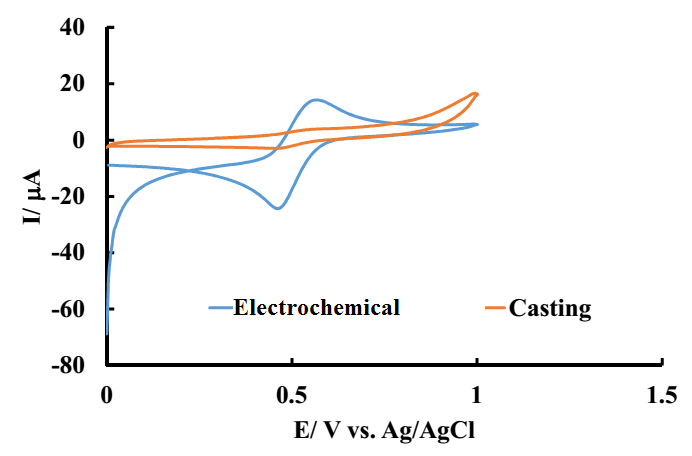


**Fig. S1.** Cyclic voltammograms of the N, S-GQD/GCE modified electrode prepared by electrochemical method or casting after dipping in 0.5 M KNO_3_ with pH 4 containing 25 µM Fe(III); Scan rate: 100 mV s^-1^.


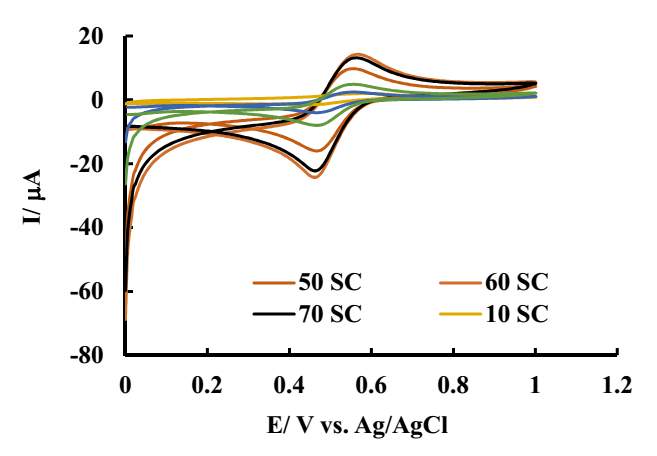


**Fig. S2.** Cyclic voltammograms of the N, S-GQD/GCE modified electrode prepare by electrochemical method using different number of cycles after dipping in 0.5 M KNO_3_ with pH 4 containing 25 µM Fe(III); Scan rate: 100 mV s^-1^.


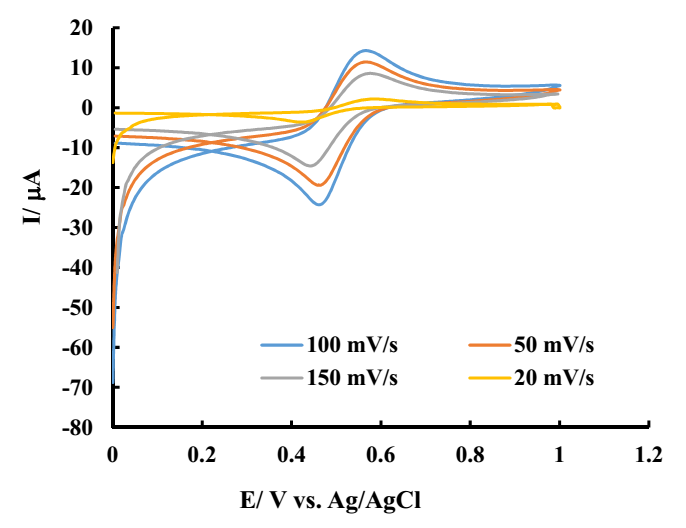


**Fig. S3.** Cyclic voltammograms of the N, S-GQD/GCE modified electrode prepared by electrochemical method using different scan rates after dipping in 0.5 M KNO_3_ with pH 4 containing 25 µM Fe(III); Scan rate: 100 mV s^-1^.


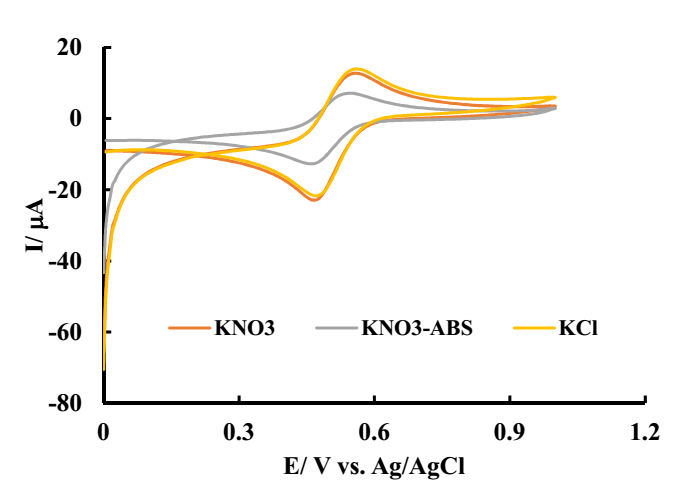


**Fig. S4.** Cyclic voltammograms of the N, S-GQD/GCE modified electrode after dipping in 0.5 M of different supporting electrolytes with pH 4 containing 25 µM Fe(III); Scan rate: 100 mV s^-1^.


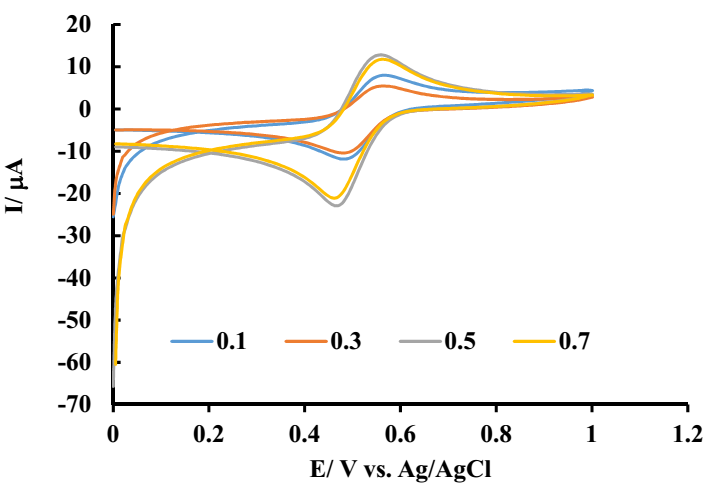


**Fig. S5.** Cyclic voltammograms of the N, S-GQD/GCE modified electrode after dipping in different concentrations of KNO_3_ with pH 4 containing 25 µM Fe(III); Scan rate: 100 mV s^-1^.


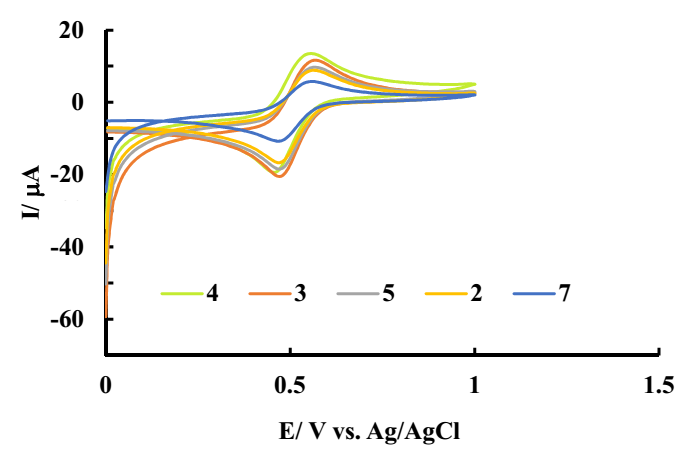


**Fig. S6.** Cyclic voltammograms of the N, S-GQD/GCE after dipping in 0.5 M KNO_3_ with different pH values containing 25 µM Fe(III); Scan rate: 100 mV s^-1^.


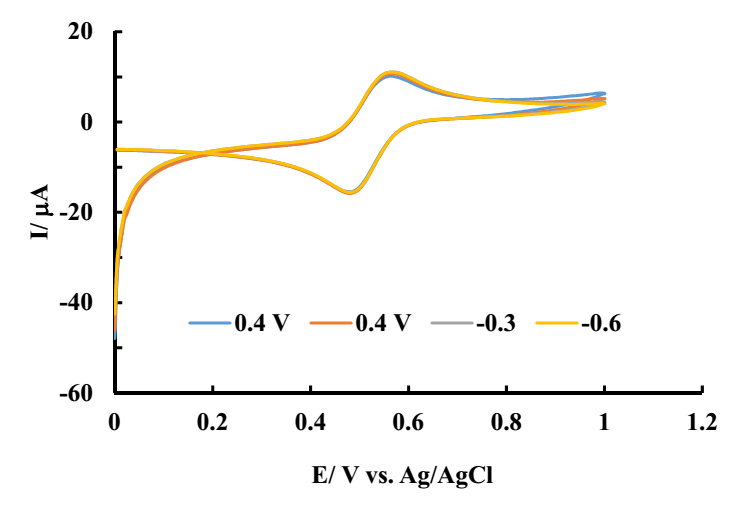


**Fig. S7.** Cyclic voltammograms of the N, S-GQD/GCE after dipping in 0.5 M KNO_3_ with pH 4 containing 25 µM Fe(III) and applying different pre-concentration potentials for 600 s; Scan rate: 100 mV s^-1^.


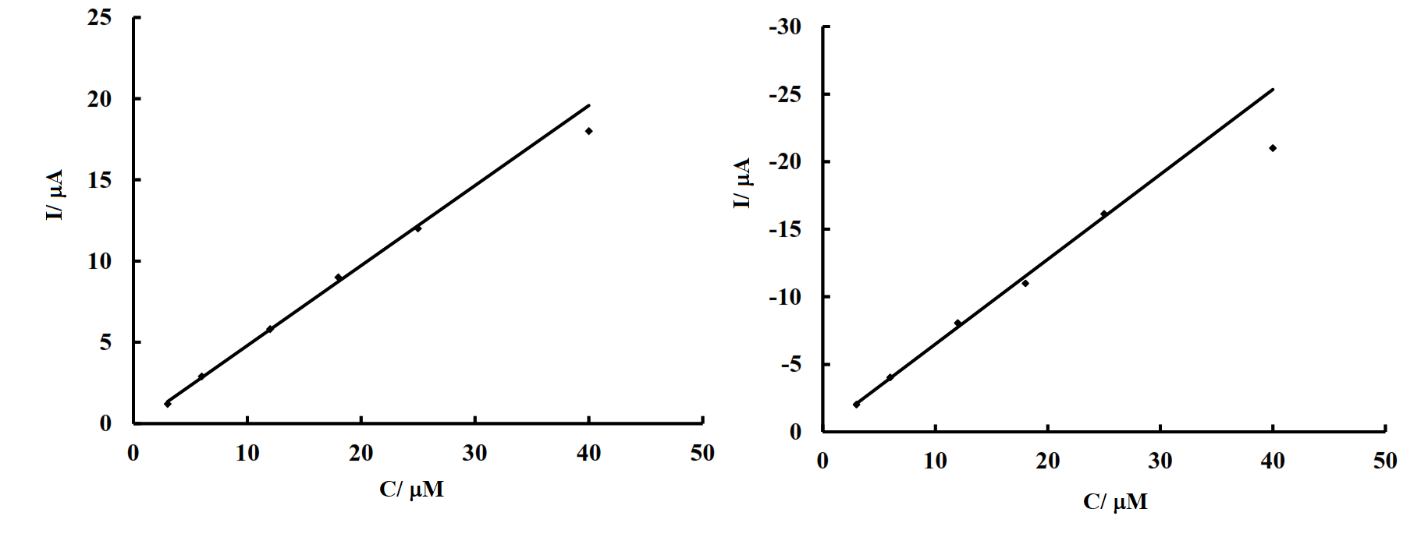


**Fig. S8.** Variation of oxidation and reduction peak currents versus concentration of Fe(III) obtained from cyclic voltammograms of the N, S-GQD/GCE after dipping in 0.5 M KNO_3_ with pH 4 containing different concentrations of Fe(III).


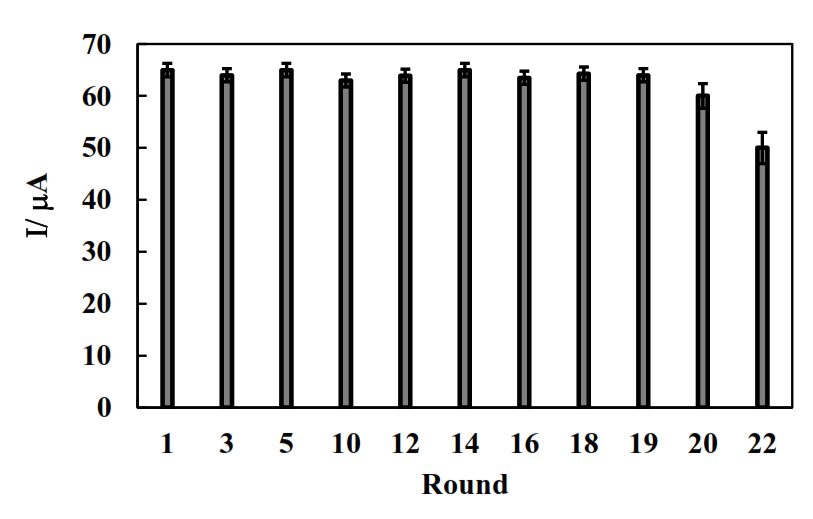


**Fig. S9**. SWV response of N, S-GQD/GCE dipped in 0.5 M KNO_3_ with pH 4 containing 80 nM Fe(III) in different repetitions .


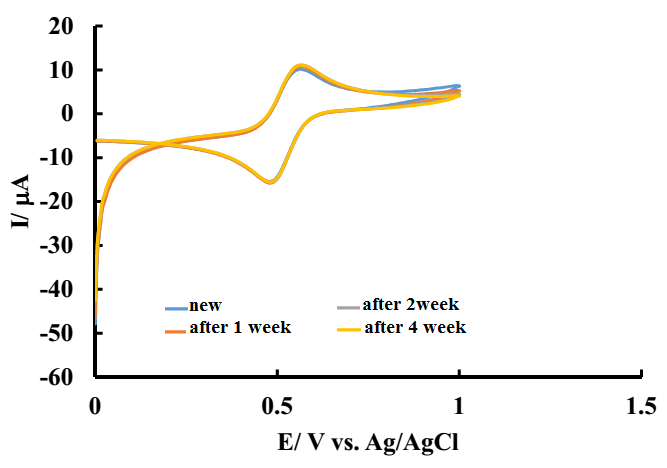


**Fig. S10.** Variation of CV signal of N, S-GQD/GCE after immersion in 25 μM of Fe(III) ions using newly prepared electrode and after storage for one, tow and four weeks; Scan rate: 100 mV s^-1^.


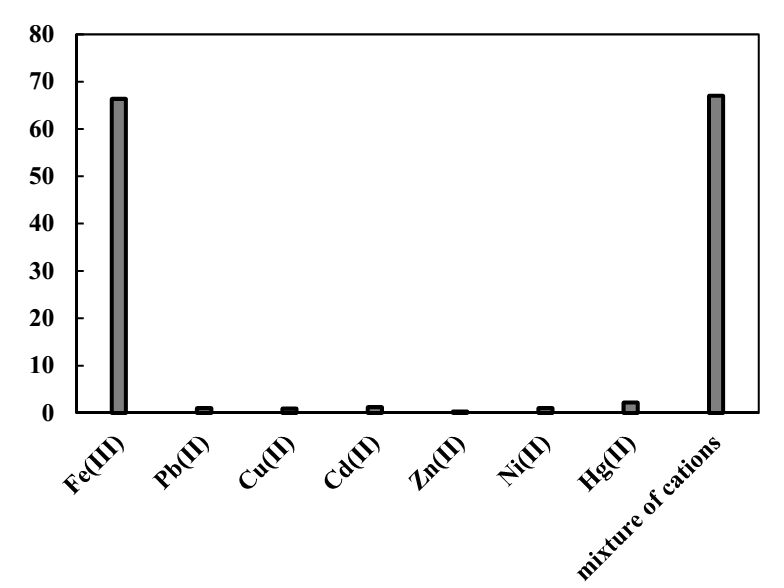


**Fig. S11**. SWV response of N, S-GQD/GCE dipped in 0.5 M KNO_3_ with pH 4 after addition of different cations with concentration 80 nM as interfering ion and a mixture of these cations.
